# COVID-19 Outcomes in Patients Undergoing B Cell Depletion Therapy and Those with Humoral Immunodeficiency States: A Scoping Review

**DOI:** 10.20411/pai.v6i1.435

**Published:** 2021-05-14

**Authors:** Jessica M. Jones, Aiman J. Faruqi, James K. Sullivan, Cassandra Calabrese, Leonard H. Calabrese

**Affiliations:** 1 Cleveland Clinic Lerner College of Medicine of Case Western Reserve University, Cleveland, Ohio; 2 Cleveland Clinic, Department of Rheumatic and Immunologic Diseases, Cleveland, Ohio

**Keywords:** COVID-19, B-cell, inborn errors of immunity, Scoping Review, Antibodies, CD20

## Abstract

**Background:**

The role of humoral immunity has been well established in reducing infection risk and facilitating viral clearance in patients with COVID-19. However, the relationship between specific antibody responses and severity of COVID-19 is less well understood.

**Methods:**

To address this question and identify gaps in knowledge, we utilized the methodology of a scoping review to interrogate risk of infection and clinical outcomes of COVID-19 in patients with iatrogenic and inborn humoral immunodeficiency states based on existing literature.

**Results:**

Among patients with iatrogenic B-cell depletion, particularly with agents targeting CD20, our analysis found increased risk of severe COVID-19 and death across a range of underlying disease states. Among patients with humoral inborn errors of immunity with COVID-19, our synthesis found that patients with dysregulated humoral immunity, predominantly common variable immunodeficiency (CVID), may be more susceptible to severe COVID-19 than patients with humoral immunodeficiency states due to X-linked agammaglobulinemia and other miscellaneous forms of humoral immunodeficiency. There were insufficient data to appraise the risk of COVID-19 infection in both populations of patients.

**Conclusions:**

Our work identifies potentially significant predictors of COVID-19 severity in patients with humoral immunodeficiency states and highlights the need for larger studies to control for clinical and biologic confounders of disease severity.

## INTRODUCTION

The host immune response to SARS-CoV-2 infection is complex and involves integration of both innate and humoral limbs [1, 2]. Much attention has been focused on humoral immunity with attempts to define its clinical importance in both protecting the host from infection as well in resolving disease [2, 3]. Clinical evidence supporting the effectiveness of humoral immunity to SARS-CoV-2 in these tasks include the therapeutic effects of monoclonal antibodies in facilitating the clearance of virus, particularly in those with suboptimal baseline humoral responses [4], and the strong correlation between the presence of baseline natural antibody status and reduced risk of reinfection [5]. The development of neutralizing antibodies has also been correlated with protection of *de novo* infection in those successfully immunized [6]. Less clear, however, is the role of specific antibody responses in recovery from COVID-19, as most investigations have demonstrated higher antibody levels in those with more severe forms of disease [7], suggesting that neutralizing antibodies may have a relatively limited impact on disease resolution.

Dissecting the precise role of humoral immunity to a viral infectious agent is daunting given that specific antibodies play numerous and interrelated roles within the integrated immune defense network [8, 9]. Beyond their capacity to block viral entry, antibodies provide defense by interacting with complement and Fc receptors on a wide variety of cells; these functions have been linked to resolution of many infectious diseases [10–15]. Under other circumstances, however, specific antibodies can enhance pathology [16], and thus understanding this balance is important as we craft more effective therapeutics and vaccines.

Among the tools to help dissect and analyze the physiologic role of specific components of the immune response in humans is the examination of clinical settings where there are selective deficiencies and appraising the outcomes in the interactions with pathogens. These deficiencies can be primary, as observed in patients with inborn errors of immunity (IEI) or iatrogenic as in patients who are treated with targeted therapies directed against discrete components of the immune response [17]. The aim of this scoping review is to systematically map the empiric evidence regarding the severity of COVID-19 in patients with these deficiency states as well as to identify any existing gaps in knowledge. Scoping reviews are a type of knowledge synthesis that follow specific methodology to summarize concepts, trends, gaps, and the variety of studies in a given field [18]. A scoping review was identified as the most appropriate method of knowledge synthesis as it was anticipated we would encounter substantial heterogeneity of study populations within these 2 broad categories as well as variability of reporting of immunologic data and outcomes. This review was designed to inform the field about the relative importance of humoral immunity in the integrated defense network with the prospects for better managing and counseling of patients so afflicted as well as providing insights into therapeutic development.

## METHODS

We utilized the methodology of a scoping review in order to investigate our overarching research question: “How do innate or iatrogenic deficiencies in humoral immunity impact clinical outcomes from COVID-19?” We followed the guidelines of Preferred Reporting Items for Systemic Reviews and Meta-Analyses Extension for Scoping Reviews (PRISMA-ScR) as outlined by Tricco et al [18].

### Eligibility criteria

Eligible studies included English-language literature related to COVID-19 and patients who received B-cell depleting therapies or with humoral inborn errors of immunity (hIEI). Within the iatrogenic B-cell depletion category of our literature search, the decision was made to include case reports and case series in order to obtain a higher degree of information about patient outcomes within each medication group than could be obtained within the larger cohort studies. In contrast, for the hIEI literature search, case reports, small-scale literature reviews, and cohort studies were included to identify all unique patients to date given the small population of hIEI patients who were infected with SARS-CoV-2.

### Information sources

For iatrogenic B-cell depletion articles, an initial PubMed search was conducted on November 18, 2020 for articles on COVID-19 (Concept 1, Supplemental data) outcomes in patients receiving CD19- and CD20-targeting medications (Concept 2, Supplemental data). Throughout the process of reviewing papers, relevant references within initial search results were also identified and included. During the writing process, select authors within the field also directed us to unpublished results. For hIEI articles, we conducted a PubMed search for articles on COVID-19 (Concept 1, Supplemental data) outcomes in patients with hIEIs through March 24, 2021. Keywords for hIEIs were systematically chosen using the 2019 Update of the IUIS Phenotypical Classification of Predominantly Antibody Deficiencies (Concept 3, Supplemental data). A final PubMed search was performed for all concepts on March 25, 2021, newly reported series within the iatrogenic subgroup, and single reports within the IEI subgroup were then analyzed and incorporated where appropriate.

### Selection of sources of evidence

All articles were first screened by title and abstract for relevance, and studies not meeting inclusion criteria were removed. Studies were then reviewed in full, and mined for type of article, and reported outcomes.

### Data charting process and data items

For all included studies, the highest level of care (outpatient, hospital ward, intensive care unit [ICU]) and clinical outcome (recovery or death) were tabulated. Parameters unique to iatrogenic B-cell depletion articles that were charted included specific B-cell depleting therapy, time from last infusion, duration of symptoms, and serology status after recovery. Levels of B cell, IgG, and IgM prior to infection were also queried, but were missing in the majority of papers and so were not included in the final report. Parameters unique to hIEI articles that were charted included demographic information (age, sex), specific diagnosis or genetic mutation, and comorbid medical conditions.

## RESULTS

### Selection and characteristics of sources of evidence

Our iatrogenic B-cell depletion search yielded 103 results. Twelve articles were removed after being deemed unrelated through screening of title and abstract. An additional 40 articles were excluded for wrong study type or irrelevant outcomes. The final analysis consisted of 51 studies: 14 cohort studies and 37 small case series or case reports. Our hIEI search yielded 66 results. Opinion articles, ecologic studies, and articles unrelated to COVID-19 hIEI clinical outcomes were excluded. Eleven articles were included for final analysis including 6 case reports [19–24], 4 cohort studies [25–28], and 1 literature review of case reports [29]. Duplicate patient reports across studies were accounted for, and only patients with clearly defined hIEIs from cohort studies were included. Table 1 lists a summary of the papers included in this scoping review by condition studied.

**Table 1:** Summary of papers included within Scoping Review

Condition Studied	Number of studies	Number of patients
Rheumatologic	9	348
Multiple Sclerosis or related	20	2,951
Hematologic	10	24
Nephrotic	3	161
Vasculitis	9	9
hIEI	11	82
Totals:	62	3,575

**Section A. Do patients with compromised humoral immunity from iatrogenic B-cell depletion therapy have greater risk of COVID-19 infection compared to the general population?**

### Epidemiologic studies of incidence without comparators

Six studies (4 in patients with multiple sclerosis [MS], 1 in a patient with rheumatic disease, and 1 in a patient with pediatric nephrotic syndrome) identified the incidence of SARS-CoV-2 infection among patients receiving B-cell depleting therapies (Table 2). The incidence rates of 4 of these studies ranged from 0-33%, and all data was collected between April and May of 2020. None of the studies provided direct comparisons to incidence rates within the general population at the time of the study data collection.

**Table 2:** Incidence of COVID-19 among patients receiving CD20 depleting drugs

Disease	Drug	# Pts.	Infection rate	Relative risk/odds ratio (Infection)	Mortality rate	How positivity was determined	Date of data extraction	Ref.
*Studies not including a comparison group*								
Multiple Sclerosis	Rituximab	54	7 (12.9%)		0	Symptoms	Apr-20	[73]
Multiple Sclerosis	Ocrelizumab	6	2(33%)		0			
Multiple Sclerosis	Ocrelizumab	6	0			Patient interview	Apr-20	[74]
Pediatric Nephrotic Syndrome	Anti CD20	159	0			Symptoms	Apr-20	[75]
Rheumato-logic Diseases	Rituximab	76	13 (17.1%)		3 (23.1%)	Symptoms	May-20	[76]
*Studies including a comparison group*								
Multiple Sclerosis	Rituximab	285	21 (7.4%)	RR: 3.55 (CI: 1.45, 8.68)^a^		Symptoms	Apr-20	[30]
Multiple Sclerosis	Ocrelizumab	12	0					
Multiple Sclerosis	Rituximab	1858	38 (2.0%)	OR:1.85 (CI: 1.37-2.33)^b^	2 (5.3%)	Positive PCR result or compatible lung CT scan	May-20	[31]
Multiple Sclerosis	Ocrelizumab	24	1 (4.2%)	OR=2.83 (CI: .81-4.84)^b^	0			

a compared to patients receiving non-cell depleting, non-cell trafficking inhibitor disease modifying therapies

b compared to patients with same disease receiving non-B cell depleting therapies

### Epidemiologic studies of incidence of SARS-CoV-2 infection with comparators

Two studies of patients with multiple sclerosis compared patients with B-cell depletion therapy risk of SARS-CoV-2 infection to patients with the same disease not receiving B-cell depleting therapies (Table 2). Both studies found an increased risk of infection among their patients receiving B-cell depleting therapies compared to those not receiving B-cell depleting therapies [30, 31]. No studies of patients with rheumatic disease or nephrotic syndrome included a comparator group not receiving B-cell depletion therapy.

Two additional studies in persons with multiple sclerosis (PwMS) reported comparisons between rates of anti-CD20 use among SARS-CoV-2 positive and negative patients. A retrospective study in Italy of 844 PwMS with suspected (n=565) or confirmed (n=279) COVID-19 found that PwMS with a suspected or confirmed SARS-CoV-2 infection were treated with ocrelizumab at a significantly higher frequency than the general Italian PwMS population (OR=1.84, 95% CI 1.31–2.56) [32]. Similarly, a study from New York of SARS-CoV-2 positive/suspect positive PwMS noted a relatively high proportion of SARS-CoV-2 infected patients receiving anti-CD20 therapies (44.7%) compared with their PwMS population, in which 33.1% of patients take anti-CD20 therapies [33]. Although these studies are limited by lack of appropriate comparison groups and adjustments for potential confounding variables, they nevertheless suggest that iatrogenic B-cell depletion may increase risk of COVID-19 infection at least within certain disease groups.

**Section B: Are patients with compromised humoral immunity at risk for more severe outcomes from COVID-19 than patients with functioning humoral systems?**

### Iatrogenic B-cell depletion – Effects on COVID-19 disease severity

Through case reports, we identified 54 individual cases of COVID-19 among patients receiving anti-CD20 medications (Supplementary Table). Among this cohort, 57% were hospitalized (31/54), 20% were treated as outpatients (11/54), 19% were treated in the ICU (10/54), and 4% did not report a severity level (2/54). Average duration of symptoms was 28.8 days.

Among case series assessing severity (Table 3), 4 clinical case series (2 in PwMS and 2 in patients with rheumatic disease) examined the severity of COVID-19 among patients receiving B-cell depleting therapies compared to patients not receiving B-cell depleting therapies. Odds ratio of severe infection ranged from 2.37 (1.18–4.74) [32] and 4.34 (1.77–10.63) [34] compared to patients with the same disease not receiving B-cell depleting therapies. Among studies examining patients with rheumatic disease, a recent series from a French registry of patients found, using multivariable analysis, that rituximab use was associated with an increased risk of severe infection (defined as requiring ICU admission or death) compared to mild or moderate (defined as requiring hospital admission) (OR=4.34, 1.77–10.63) [34]. Among this same cohort, a later study utilizing propensity scoring to adjust for confounders found that rituximab users more frequently had severe disease, (OR=3.26, 95% CI 1.66–6.40) and longer duration of hospital stay (HR=0.62, CI 0.46–0.85) than non-rituximab users [35]. Among the studies of PwMS, Sormani et al [32] found an increased risk of severe disease (defined as developing at least 1 of death, ICU admission, diagnosis of pneumonia, or hospitalization) among patients receiving the anti-CD20 agents ocrelizumab or rituximab compared to PwMS with COVID-19 not receiving B-cell depleting therapies, adjusting for likely cofactors that could affect disease outcome (OR= 2.37, 1.18–4.74) [32]. A North American cohort of PwMS found that even after adjusting for confounders, patients receiving rituximab had 4.5-fold increased odds of hospitalization for COVID-19 (2.10–9.90) but no change in ICU or ventilatory support need, compared with those not taking any disease modifying therapy (DMT). Furthermore, ocrelizumab also increased odds of hospitalization, but to a lesser degree (OR= 1.63, .98–2.72) [36].

**Table 3:** Mortality Rate among COVID-19+ patients receiving B-cell depleting therapies

Disease	Drug	# Pts.	Mortality rate	Odds Ratio (Risk of mortality)	How positivity was determined	Date of data extraction	Ref.
*Studies without a comparison group for mortality*							
Rheumatic Diseases	Anti CD20	3	1(33%)		Unknown	Unknown	[77]
Rheumatic Diseases	Rituximab	7	1 (14.3%)		Nasopharyngeal swabs or symptoms with compatible lung imaging and/or positive serology	Jun-20	[78]
Multiple Sclerosis	Anti CD20	34	2 (5.9%)		Health care provider	Apr-20	[33]
Multiple Sclerosis	Rituximab	5	1 (20%)		Symptoms	May-20	[32]
Multiple Sclerosis	Ocrelizumab	83	1 (1.2%)				
Cancer	Anti CD20	14	1 (7.1%)		SARS-CoV2 RT-PCR	Apr-20	[79]
*Studies including a comparison group for mortality*							
Rheumatic Diseases	Rituximab	192	42 (21.9%)	OR=4.04 (2.32-7.03)^a^	Physician Report	Jul-20	[37]
Rheumatic Diseases^b^	Rituximab	34/63	7 (20.6%)/13(21%)	OR=4.04 (1.35-12.04^c^ / OR=1.32 (0.55-3.19)^d^	Physician Report	May-20/Nov-20	[34] / [35]
Multiple Sclerosis	Rituximab	77	3(3.9%)	OR=2.81 (0.45-17.70)^e^	Physician Report	Dec-20	[36]
Multiple Sclerosis	Ocrelizumab	484	11(9.1%)	OR = 0.47 (0.17-1.30)+			

a Compared to patients taking methotrexate

b Two studies using the same cohort were identified. Each study sample and findings are reported within this row.

c Cannot determine how the comparison group was defined. Adjusted for age and sex

d Compared to non-rituximab users. Propensity score weighted adjustment for multiple covariates.

e Compared to no disease modifying therapy. Multivariable multinomial logistic regression model adjusting for age, sex, race, ambulation, cigarette smoking, glucocorticoid use, comorbidities, and DMTs.

In terms of variables reflecting disease severity, death is the most specific. Ten case series were identified that provided mortality rates among patients receiving CD20 depleting therapies with COVID-19 or suspected COVID-19 (Tables 2 and 3). Among these, 5 studies focused on PwMS, 4 on patients with rheumatic diseases, and 1 on a patient with cancer (Tables 2 and 3). The number of patients receiving B-cell depleting drugs in these series ranged from 3 to 1858 and the mortality ranged from 0 to 33% (Tables 2 and 3).

Two studies of rheumatic diseases incorporated multivariable logistic regression analysis and a comparator group to assess risk of mortality among those with rheumatic disease receiving B-cell depleting therapy (Table 3) [34, 37]. In the French registry, the odds ratio for death among patients receiving rituximab compared to a matched control group was elevated (4.04, 1.35–12.04) [34]. This cohort was reanalyzed in a later study with a larger sample size using propensity scoring and found that the adjusted risk of death was not significantly increased in the rituximab group compared to non-rituximab users (OR=1.32, 95% CI 0.55–3.19) [35]. In the largest study of patients with rheumatic disease and COVID-19, the Global Rheumatology Alliance analyzed 3729 patients (192 of whom were receiving rituximab) and found rituximab carried the highest odds of death compared to patients taking methotrexate monotherapy in both unadjusted and adjusted multivariable logistic regression models (OR=4.04, 2.32–7.03) [37].

The North American Registry by Salter et al also looked at mortality among PwMS receiving B-cell depleting therapies. They found no statistically significant difference in risk of mortality between patients receiving rituximab or ocrelizumab therapy compared to patients not receiving DMT after multivariable adjustment [36]. Thus, based upon these data, even with their inherent limitations, it appears that iatrogenic B-cell depletion may be associated with both increased risk of severe disease and risk of mortality among patients both with rheumatic disease as well as MS.

### Iatrogenic B-cell depletion — Effects on response to COVID-19 therapies

At time of writing, only a single study was found investigating treatment for COVID-19 infection specifically for B-cell depleted patients. Seventeen patients receiving B-cell depleting therapy (nearly all of whom had a profound hypogammaglobulinemia associated with an absence of circulating B cells, and none had mounted a neutralizing antibody response after several weeks of symptoms) were treated with convalescent plasma, which led to striking improvement of clinical symptoms and biological parameters in 16 out of 17 patients and a decrease of SARS-CoV-2 RNAemia within 7 to 14 days, highlighting the potential importance of circulating antibodies in infection clearance [38].

### Humoral Inborn Errors of Immunity — Effects on COVID-19 disease severity

Our search identified 82 unique patients (34% female) with hIEIs and confirmed SARS-CoV-2 infection from across the globe (Table 4). The majority of patients (49) had common variable immunodeficiency (CVID) [19, 22–29]. Fifteen patients had agammaglobulinemia (12 X-linked, 3 autosomal recessive) [21, 25–29]. Six patients had hyper-IgM disease [25, 27, 28]. Five patients had specific immunoglobulin deficiencies (IgG, IgA, or both) [20, 25–27]; 4 patients had otherwise unspecified hypogammaglobulinemia [25, 26]; 2 patients had antibody deficiency with syndromic features [26]; and 1 patient had activated PI3K delta syndrome (APDS) [26]. The age distribution of the cohort ranged from pediatric patients to the elderly (>75 years), though patients with agammaglobulinemia were younger on average than their CVID counterparts (no patient older than 54). Mean age could not be calculated due to incomplete data.

**Table 4:** Clinical Outcomes in Patients with hIEI and with Confirmed COVID-19

Immunodeficiency	# of Patients (# Females)	Age Range	# Outpatient*	# Hospital Ward*	# ICU*	# Recovered	# Died	Ref.
CVID	49 (21)	8 to 75+	20	17	12	41	8	[19, 22–29]
XLA	12 (0)	5 to 54	3	9	0	12	0	[25–29]
ARA	3 (0)	35 to 64	2	0	1	3	0	[25, 26]
Hypogamma, unspecified	4 (3)	3 to 75+	2	1	1	3	1	[25, 26]
Ig Deficiency	5 (2)	8 to 75+	1	1	3	3	2	[20, 25, 26]
HIGM	6 (1)	6 to 30	4	2	0	6	0	[25, 27, 28]
Syndromic Ab deficiency	2 (0)	3-12; 35 to 44	0	1	1	1	1	[26]
APDS PIK3R1	1 (1)	25-35	1	0	0	1	0	[26]
TOTAL	82 (28)		33	31	18	70	12	

CVID: Common Variable Immunodeficiency; XLA: X-linked Agammaglobulinemia; ARA: Autosomal Recessive Agammaglobulinemia; HIGM: Hyper-IgM Syndrome; APDS PIK3R1: Activated Phosphoinositide 3-Kinase δ Syndrome

The risk of SARS-CoV-2 infection among patients with hIEI remains incompletely understood, as most studies examined clinical outcomes in hIEI patients with confirmed SARS-CoV-2 infection rather than incidence of infection. However, 1 large cohort study of 4718 patients with primary immunodeficiencies (PIDs) in Iran, including 1001 with hIEIs alive during the pandemic, found 19 confirmed cases of SARS-CoV-2 infection, with 4 cases among hIEI patients. Overall, the incidence of infection in this PID cohort was only 1.23-fold higher than the general population. Importantly, as the authors noted, the external validity of this may be limited due to pediatric skew of the cohort as well as increased precautions taken by patients with PIDs [27].

Within the CVID cohort (43% female) identified by our search, 12 patients (24.5%) required intensive care. Seventeen patients were hospitalized without intensive care, and the 20 remaining patients were either asymptomatic or received outpatient care only. Treatment regimens varied from supportive care only to aggressive multidrug regimens of antibiotics, steroids, and immunomodulatory agents. Patient comorbidities also varied significantly, from none to chronic lung, liver, endocrine, cardiovascular, and kidney disease. In total, 8 (16.3%) CVID patients died, 7 of them female. Among 15 CVID patients without notable comorbidities, 1 died, and among 7 patients age 65 or older, 2 died [19, 22–29] termed coronavirus disease 2019 (COVID-19).

Among 15 agammaglobulinemia patients (100% male), 1 patient required intensive care, 9 patients were hospitalized without intensive care, and 5 patients were either asymptomatic or received outpatient care only. Treatment varied from supportive care only to convalescent plasma infusions, antibiotics, and immunomodulatory agents. Strikingly, although nearly half (7) of these patients had pre-existing comorbid lung disease (eg, COPD, bronchiectasis), all of them recovered from COVID-19 [21, 25–29].

Based on these limited data, it appears that patients with dysregulated humoral immune responses, such as CVID, may be more susceptible to severe COVID-19 than patients with ostensibly more severe, but select, humoral immunodeficiency states characterized by agammaglobulinemia.

## DISCUSSION

To the best of our knowledge, this scoping review is the first attempt to analyze and synthesize the role of humoral immunity in COVID-19 infection across a spectrum of clinical disorders caused by iatrogenic B-cell depletion and inborn errors of immunity. We believe our observations have potentially important implications for understanding the network of host defense against SARSCoV-2, but have significant limitations in their strength, as will be discussed.

Defining the precise role of humoral immunity in the integrated defense against SARS-CoV-2 infection is problematic and incompletely understood. Antibodies (specifically IgG, IgM, and IgA) have long been recognized as important components of adaptive immune defense and protection in respiratory viral infections, particularly in influenza and other human coronaviruses [8, 10, 13]. In COVID-19, there is now clear evidence that a SARS-CoV-2 specific antibody response is important during the early stages of infection, evidenced by the strong correlation between antibody response to vaccine and protection from incident and severe infections in non-human primate studies [39, 40], the effectiveness of monoclonal antibodies [41] and convalescent plasma [42] early in infection, and the correlation between the neutralizing antibody seroconversion and multiple log reduction in viral load in cases of uncomplicated infection in humans [41].

The role of specific antibodies in later stages of the infection is less clear, as most patients with advanced forms of COVID-19 have higher viral loads than those with mild and early disease despite higher levels of antibodies with neutralizing capacity [43, 44]. Further, the same monoclonal antibody therapy effective at controlling infectious spread early in COVID-19 is not effective in later stages of disease [4].

The results of our scoping review provide insights from 2 disease models that both share deficits of humoral immune responsiveness: (1) iatrogenic depletion of B cells with biologic therapies and (2) primary inborn errors of immunity with explicit humoral defects. Each provides some degree of insight into the role of antibodies against SARS CoV-2.

From our examination of COVID-19 in patients with IEIs, we found no studies of adequate design to assess whether patients are more susceptible to SARS-CoV-2 infection. Largely from detailed individual case reports and small series (Table 4), we did appraise evidence documenting recovery from SARS-CoV-2 infection without a significant antibody response. This evidence confirms a major role for cell-mediated immunity in infection resolution [45] and is consistent with numerous reports of recovery in healthy individuals without generating detectable antibody response [7, 46].

Within the IEI spectrum, our review supports a general trend for greater morbidity and mortality among patients with CVID than patients with X-linked agammaglobulinemia (XLA), an observation previously made by others [26, 29, 47]. The reasons for this differential disease severity between these subsets of IEI are unclear but several factors deserve comment. First, CVID is a highly heterogenous immune deficiency disorder which, by definition, includes defective humoral immune function, but often is attended by variable defects within the cell-mediated immune compartment as well [48, 49].

In contrast, patients with XLA display defective B-cell maturation with relatively preserved T-cell function as reflected by relatively preserved *ex vivo* responses to respiratory viruses [50]. Severe COVID-19 disease is often accompanied by a state of hyperinflammation, raising the question of whether patients with CVID, who are also at elevated risk of dysregulated inflammatory and granulomatous reactions [51–53], may be predisposed to such immunodysregulation. In CVID, cellular abnormalities in T-cell compartments (ie, reduced T-regulatory cells [Tregs] and increased T-follicular helper cells [Tfh], along with perturbations of B-cell subsets and increases in innate lymphoid type 3 cells) may all contribute to immunodysregulation and promote a hyperinflammatory milieu [54, 55].

An additional mechanistic explanation for the favorable survival rate among those with XLA may be related to the expression of Bruton's Tyrosine Kinase (BTK) in macrophages and its role in TLR-mediated NF-*k*B triggering of the production of multiple cytokines incriminated in the hyperinflammatory phase of COVID-19 [56]. Based on this rationale, the covalent inhibitor of BTK acalbrutinib has been used in a small open-label trial of 19 patients with severe COVID-19 demonstrating some degree of clinical success as well as *ex vivo* evidence of elevated BTK activity [57]. Detailed investigations attempting to understand the immunopathogenesis of COVID-19 in patients with humoral immunodeficiency states to date have been limited, but a recent study performing comprehensive flow cytometry examining subsets of T and B cells in a single patient with CVID and an immunocompetent control who both recovered from COVID-19 revealed a series of often contrasting abnormalities in both T-cell and B-cell compartments between experimental subjects [58]. The remaining forms of humoral IEI encountered in our review are too rare to draw any further conclusions regarding outcomes, though 13/17 (76%) with these miscellaneous conditions did survive (Table 4).

Lastly, the patients with CVID included in this review tended to be older and had a greater burden of comorbidities, such as chronic lung disease. As we were unable to adjust for demographic differences between these 2 cohorts in this scoping review due to non-uniform reporting, it is possible these factors, particularly age, also underlie the observed differences in COVID-19 severity and survival. Consistent with this, a recent study from the United Kingdom demonstrated better outcomes in XLA patients, with zero reported deaths among 4 patients (mean age 30 years), compared to CVID patients, with 8 reported deaths among 23 patients (mean age 54 years) [54]. However, our observation that all agammaglobulinemia patients with comorbid lung disease recovered from COVID-19 suggests that the stark differences in outcomes between CVID and agammaglobulinemia patients may at least be partially attributable to immunologic determinants and cannot be entirely explained by comorbidities. Additional work, including the establishment of detailed prospective registries for the hIEI population capturing demographics, immunologic variables, and comorbidities will be needed to further clarify this issue.

Our review of patients with iatrogenic B-cell depletion also revealed no studies of sufficient rigor to assess whether patients receiving such therapies are more predisposed to SARS-CoV-2 infection. In terms of disease severity in patients with iatrogenic B-cell depletion states, our review suggests that such patients are at a higher risk for severe outcomes, including death, across underlying disease states (ie, rheumatic disorders, multiple sclerosis) (Tables 2 and 3). Although severe outcomes can potentially be attributed to numerous associated variables, such as underlying disease states, comorbidities, age, and other therapies, our review identified several studies of large numbers of patients [32, 34, 37] where multivariable analysis to control for such confounding was performed, adding weight to the idea that iatrogenic B-cell depletion itself may be driving risk. Though the registries on which these data are based are limited by reporting bias and low granularity of data collection, these effects merit continued monitoring as patient registries grow.

While conclusions of increased risk of severe COVID-19 with B-cell depletion are cautionary, they raise a number of questions as to what may underlie these biologic effects. Patients receiving B-cell depleting therapies are well documented to be at risk for serious infections [59], but the effects differ across indications (rheumatic, MS, hematologic etc.) [60–62]. Among our reported studies of COVID-19 infection, mortality and severity rates varied among different diseases. Whether quantitative or functional deficits of immunoglobulins contributed to these effects is of interest, but levels were not reported in the cohort studies included in our analysis. A recent report from the Roche/Genentech clinical trial data of PwMS treated with ocrelizumab found that, among those with COVID-19 with previous serum immunoglobulin levels reported (27/51, 52.9%), all had IgG levels within the normal range [63].

This finding should be regarded with caution due to the small sample size and lack of statistical modeling. However, in general, immunoglobulin levels are usually normal, even in the setting of long-term B-cell depleting therapies, though approximately 25% may ultimately have reduced levels of 1 or more immunoglobulin isotype [59]. Furthermore, it is well established that patients treated with B-cell depleting therapy have compromised humoral immunity with profound peripheral B-cell depletion, generally lasting 6-9 months or longer [64]. Unfortunately, B-cell levels were not reported in the majority of studies reviewed, and we were unable to analyze the direct correlation between B-cell levels and COVID-19 infection. Additionally, patients receiving B-cell depleting therapies experience impairments in their ability to generate normal antibody responses to a variety of vaccine challenges (ie, T-cell dependent, T-cell independent, and neoantigens) [64–66], yet can generate cell-mediated immune responses to the recombinant zoster vaccine [67]. How patients undergoing B-cell depletion therapy will respond to COVID-19 vaccines is a particularly pressing issue; prospective studies examining humoral and cellular responses as well as tracking clinical effectiveness of vaccination to SARS-CoV-2 among this population are urgently needed.

The conclusions of this scoping review should be considered in the context of its limitations. For both categories of humoral immune deficiency reviewed, a lack of granularity and completeness of data including demographics, co-morbidities, immunologic data, and inconsistent definitions of severe COVID-19 were problematic. In particular, the reports in patients receiving B-cell depleting agents were essentially devoid of critical immunologic data such as immunoglobulin levels and B-cell numbers. We attempted to bridge this gap through case reports, but we were unable to consistently report immunoglobulin or B-cell levels in these patients. Another particularly important consideration within this patient population is timing of infection in relationship to last treatment. This latter point is of particular importance given the pharmacokinetics and pharmacodynamics of B-cell depleting monoclonal antibodies on B-cell depletion and reconstitution, and thus such data are critical for understanding the putative effects on COVID-19 outcomes. In their Italian PwMS population, Sormani et al discovered no association between COVID-19 severity and the time passed since the last anti-CD20 infusion (OR=2.77, 95% CI 1.31–5.89 for last infusion within 3 months and OR=2.05, 95% CI 0.97–4.28 for last infusion before 3 months). Interestingly, they did report a trend of increased severity with increased duration of anti-CD20 therapy [32]. An analysis of the French Registry rituximab cohort found that reduced time from last infusion to COVID-19 infection was associated with more severe disease and higher risk of death [35].

Another limitation of most of the reported series of patients receiving B-cell depleting agents was a lack of well-defined comparison groups and inconsistent definitions of severe COVID-19, thus limiting our ability to interpret their potential confounding effects in these patient populations (eg, age, other pulmonary comorbidities) on outcomes. Thus, more robust studies, prospectively collected with larger sample sizes and comparator groups as well as detailed case reports with more comprehensive immune response profiles are needed to identify the risk of severe disease and mortality due to COVID-19 in these patient populations and to uncover possible pathophysiologic mechanisms.

In terms of the population with hIEIs, these are rare diseases and the prevalence of primary hIEIs is low, so the number of patients included in our scoping review was limited. While many of the other hIEIs described in table 4 (such as HIGM) present an intriguing course of COVID-19, with the majority recovering without admittance to the ICU, these studies did not uniformly report on comorbidities, concomitant treatments (such as Ig replacement therapy), or specific gene mutations, and had no comparator group, representing significant potential confounders in the interpretation of clinical outcomes in this unique cohort of patients. Lastly, we were cognizant of the potential for duplicate patients in our literature search and removed apparent duplicates from the hIEI case report summary table, and reported results from the iatrogenic case reports separately from cohort studies. However, it is still possible some of the trends identified were augmented by duplicate reports.

Finally, it should be noted that this review limited its scope to immunodeficiency states primarily compromising the humoral compartment. Therefore, other immunodeficiency states attended by humoral deficits that may affect COVID-19 clinical outcomes were not evaluated. Among these other diseases and corresponding immunodeficiency states, HIV infection deserves specific comment given the significant public health implications in relationship to COVID-19 [68]. HIV was not included in our analysis as it principally targets the T-cell compartment, and we did not consider HIV patients on highly active anti-retroviral therapy (HAART) to be immunocom-promised. Nevertheless, it is well established that HIV also causes B-cell dysfunction [69], and further studies are warranted to interrogate its impact on COVID-19 clinical outcomes.

The implications of this scoping review are several. First, from several sources, we have confirmed that some patients with profound inherited and acquired deficits of humoral immunity may recover form COVID-19, but certain subgroups of these patients may be vulnerable to more severe outcomes. As described previously [70, 71] the likelihood that patients given B-cell depleting therapies will make any meaningful humoral response to COVID-19 vaccination is low, in light of their suppressed response to other T-cell dependent vaccines even when administered at their pharmacodynamic nadir [64]. However, it is unknown whether such patients can develop cell-mediated immune responses to COVID-19 vaccinations and whether such response will confer significant protection. Thus, studies examining humoral and cellular vaccine responses as well as the effects of timing vaccine administration relative to drug administration are urgently needed. The same questions apply to patients with humoral IEI. While approximately 20% of patients with CVID taking immunoglobulin replacement may serologically respond to influenza vaccine [72], the majority do not; the response to COVID-19 vaccination in patients with IEI merits future study. In total, the disease course of COVID-19 in patients with humoral immune deficiencies provides insight into both the role of humoral- and cell-mediated arms of the adaptive immune system in SARS-CoV-2 infection.

## SUPPLEMENTARY DATA

Supplementary materials are available at the *Pathogens and Immunity* website. Supplementary data may be provided by the authors to benefit the reader. Supplementary data are not copyedited and are the sole responsibility of the authors. Questions or comments related to supplementary materials should be addressed to the corresponding author.
