# Supplementary material for: COVID-19 Outcomes in Patients Undergoing B Cell Depletion Therapy and Those with Humoral Immunodeficiency States: A Scoping Review
Source: Pathog Immun. 2021 May 14;6(1):76–103. doi: 10.20411/pai.v6i1.435 (PMC8150936; doi:10.20411/pai.v6i1.435)
Supplement: Supplementary Methods [file pai-6-076-s01.pdf]

Supplemental Methods

Search Concepts:

Concept 1:
("COVID"[Text Word] OR "COVID-19"[Text Word] OR "novel corona virus"[Text Word] OR "SARS-CoV-2"[Text Word] OR "COVID-19"[Supplementary Concept])

Concept 2:
("Rituximab"[Text Word] OR "Anti-CD20"[Text Word] OR "CD20 Antibody"[Text Word] OR "ofatumumab"[Text Word] OR "ocrelizumab"[Text Word] OR "obinutuzumab"[Text Word] OR "veltuzumab"[Text Word] OR "Rituximab"[MeSH Terms])

Concept 3:
"Agammaglobulinemia"[Mesh] OR “Hypogammaglobulin*”[tiab] OR “Agammaglobulin*”[tiab] OR “X-Linked Agammaglobulin*”[tiab] OR “APDS”[tiab] OR “PTEN”[tiab] OR “ARHGEF”[tiab] OR “SH3KBP1”[tiab] OR “CIN85”[tiab] OR “SEC61A1”[tiab] OR “RAC2”[tiab] OR “CD19 deficienc*”[tiab] “CD20 deficienc*”[tiab] OR “CD21 deficienc*”[tiab] OR “CD81 deficienc*”[tiab] OR “TRNT1”[tiab] OR “NFKB1”[tiab] OR “NFKB2”[tiab] OR “IKAROS”[tiab] OR “IKZF1”[tiab] OR “ATP6AP1”[tiab] OR “MOGS”[tiab] OR “TACI deficienc*”[tiab] OR “TNFRSF13B”[tiab] OR “BAFF”[tiab] OR “TNFRSF13C”[tiab] OR “IRF2BP2 deficienc*”[tiab] OR “AID deficienc*”[tiab] OR “AICDA”[tiab] OR “UNG deficienc*”[tiab] OR “INO80”[tiab] OR “MSH6”[tiab] OR “IgA deficienc*”[tiab] OR "IgG deficienc*"[Mesh] OR “IgG subclass deficienc*”[tiab] OR “antibody deficienc*”[tiab] OR “14q32”[tiab] OR “kappa chain deficienc*”[tiab] OR “IGKC”[tiab] OR “IgM deficienc*”[tiab] OR “CARD11”[tiab] OR “primary immunodeficienc*”[tiab]

S1: Summary of Case reports of COVID-19 infection among patients with anti-CD20 therapy.

Category of disease	*Disease*	*Anti-CD20 medication*	*Time since last infusion (months)*	*Outcome*	*Severity*	*Positive serology after recovery*	*Reported days of symptoms*	*Reference*
**Hematologic**								
	Primary mediastinal large B-cell lymphoma	Rituximab	0	Recovered	Hospitalized		29	[80]
	CD20+ B cell acute lymphoblastic leukemia	Rituximab	0.25		ICU stay			[81]
	Mantle Cell Lymphoma	Rituximab	0.5	Death	Hospitalized			[82]
	Marginal zone lymphoma	Rituximab	0.5	Recovered	Hospitalized	Yes	86	[83]
	Diffuse large B cell lymphoma	Rituximab	0.5	Death	Hospitalized			[82]
	Follicular lymphoma	Rituximab	4	Still symptomatic	Hospitalized	No	51	[84]
	Chronic lymphocytic leukemia	Rituximab	6	Recovered	Hospitalized		79	[85]
	Nodal marginal zone lymphoma	Rituximab	8	Recovered	Hospitalized		24	[86]
	Non-Hodgkin lymphoma	Obinutuzumab	1	Recovered	Hospitalized	No	90	[87]
	Follicular lymphoma	Obinutuzumab	2	Recovered	Hospitalized	Yes	140	[88]
	Sub-totals	*Rituximab= 8/10 Obinutuzumab= 2/10*	*Average= 2.3*	*Recovered=6/9 Still Symptomatic=1/9 Death=2/9*	*Outpatient=0/10 Hospitalized=9/10 ICU stay=1/10*	*Yes=2/4 No=2/4*	*Average= 71.3*	
**Nephrotic**								
	Membranous nephropathy	Rituximab	1	Recovered	Hospitalized		26	[89]
	Cryoglobulinemic glomerulonephritis	Rituximab	7	Recovered	ICU stay		30	[90]

	Sub-totals	*Rituximab= 2/2*	*Average= 4*	*Recovered=2/2 Still Symptomatic=0/2 Death=0/2*	*Outpatient=0/2 Hospitalized=1/2 ICU stay=1/2*	*Yes= No=*	*Average= 28*	
**Vasculitis**								
	Granulomatosis with polyangiitis (GPA)	Rituximab		Recovered	Hospitalized		45	[91]
	GPA	Rituximab		Death	ICU stay		45	[92]
	GPA	Rituximab	0	Recovered	Hospitalized		29	[93]
	GPA	Rituximab	0.5	Recovered	Hospitalized		48	[94]
	EosiNophilic GPA	Rituximab	0.75	Recovered	Hospitalized		14	[95]
	GPA	Rituximab	1	Recovered	Hospitalized	No	40	[96]
	GPA	Rituximab	2	Recovered	Hospitalized		19	[97]
	Myeloperoxidase-ANCA microscopic polyangiitis	Rituximab	4	Recovered	Outpatient	Yes	14	[98]
	GPA	Rituximab	5	Recovered	Hospitalized	No	29	[99]
	Sub-totals	*Rituximab=9/9*	*Average= 1.9*	*Recovered= 8/9 Still Symptomatic=0/9 Death= 1/9*	*Outpatient=1/9 Hospitalized=7/9 ICU stay= 1/9*	*Yes= 1/3 No= 2/3*	*Average= 31.4*	
**Multiple Sclerosis**								
	Multiple Sclerosis	Ocrelizumab	0.25	Recovered	Outpatient		0	[100]
	Multiple Sclerosis	Ocrelizumab	1	Recovered	Outpatient	No	0	[101]
	Multiple Sclerosis	Ocrelizumab	2	Recovered	Outpatient	No	30	[102]
	Multiple Sclerosis	Ocrelizumab	3	Recovered	Hospitalized	Yes	26	[101]
	Multiple Sclerosis	Ocrelizumab	3	Recovered	Outpatient		3	[103]
	Multiple Sclerosis	Ocrelizumab	4	Recovered	Outpatient		3	[101]
	Multiple Sclerosis	Ocrelizumab	4	Recovered	Outpatient	No	2	[102]
	Multiple Sclerosis	Ocrelizumab	5	Recovered	Hospitalized	Yes	24	[104]
	Multiple Sclerosis	Ocrelizumab	5	Recovered	Outpatient	Yes	14	[105]
	Multiple Sclerosis	Ocrelizumab	6	Recovered	Hospitalized	No	19	[104]
	Multiple Sclerosis	Ocrelizumab	7	Recovered	Hospitalized			[106]
	Multiple Sclerosis	Ocrelizumab	7	Recovered	Outpatient	No	7	[101]
	Multiple Sclerosis	Ocrelizumab	7	Recovered	Outpatient	Yes	0	[101]
	Multiple Sclerosis	Ocrelizumab	14	Recovered	Hospitalized	Yes	10	[101]
	Multiple Sclerosis	Ocrelizumab		Recovered	Outpatient			[107]
	Multiple Sclerosis	Ocrelizumab		Recovered	Hospitalized		10	[108]
	Multiple Sclerosis	Rituximab	2			No		[109]

Multiple Sclerosis	Rituximab	2			No		[109]
Multiple Sclerosis	Rituximab	2	Recovered	Hospitalized	No	14	[110]
Multiple Sclerosis	Rituximab	4			No		[109]
Multiple Sclerosis	Rituximab	5	Recovered	Hospitalized		7	[111]
Multiple Sclerosis	Rituximab	7	Recovered	Hospitalized	No	16	[112]
Multiple Sclerosis	Rituximab	8	Recovered	Hospitalized		9	[101]
Sub-totals	*Rituximab= 7/23<br>Ocrelizumab=16/23*	*Average= 4.7*	*Recovered= 20/20<br>Still Symptomatic= 0/20<br>Death=0/20*	*Outpatient= 10/20<br>Hospitalized= 10/20<br>ICU stay= 0/20*	*Yes=5/15<br>No=10/15*	*Average= 10.8*	
**Neuromyelitis optica spectrum disorders**							
Neuromyelitis optica - spectrum disorders	Ofatumumab	0.33			No		[109]
Aquaporin-4-positive neuromyelitis optica spectrum disorder	Rituximab		Recovered	Hospitalized		36	[113]
Neuromyelitis optica spectrum disorder	Rituximab	5	Recovered	ICU stay	Yes	46	[110]
Sub-totals	*Rituximab= 2/3<br>Ofatumumab = 1/3*	*Average= 2.7*	*Recovered= 2/2<br>Still Symptomatic=0/2<br>Death= 0/2*	*Outpatient=0/2<br>Hospitalized=1/2<br>ICU stay=1/2*	*Yes= 1/2<br>No=1/2*	*Average= 41*	
**Rheumatologic**							
Polymyositis and Sjögren’s syndrome	Rituximab	2	Recovered	ICU stay	No	32	[114]
Polymyositis and Sjögren’s syndrome	Rituximab	2	Recovered	ICU stay		63	[114]
Rheumatoid Arthritis	Rituximab	0.5	Death	ICU stay		14	[115]
Rheumatoid Arthritis	Rituximab		Death			17	[115]
Systemic Sclerosis	Rituximab	3	Recovered	ICU stay		19	[116]
Systemic Sclerosis	Rituximab	5	Still symptomatic	ICU stay		23	[116]
Systemic Sclerosis	Rituximab	10	Still symptomatic	Hospitalized		15	[116]
Sub-totals	*Rituximab= 7/7*	*Average= 3.7*	*Recovered=3/7<br>Still Symptomatic=2/7<br>Death=2/7*	*Outpatient=0/7<br>Hospitalized=1/7<br>ICU stay=6/7*	*Yes=0/1<br>No= 1/1*	*Average= 26.1*	

**Overall totals**	***Obinutuzumab=2<br>Ocrelizumab=16<br>Ofatumumab=1<br>Rituximab=35***	***Average= 3.5<br>N=48***	***Recovered= 41<br>Still Symptomatic= 3<br>Death=5***	***Outpatient= 11<br>Hospitalized= 31<br>ICU stay= 10***	***Yes= 9<br>No= 16***	***Average= 28.8<br>N=45***
